# FunFEA: an R package for fungal functional enrichment analysis

**DOI:** 10.1186/s12859-025-06164-7

**Published:** 2025-05-27

**Authors:** Julien Charest, Paul Loebenstein, Robert L. Mach, Astrid R. Mach-Aigner

**Affiliations:** Institute of Chemical, Environmental and Bioscience Engineering, TU Wien, Gumpendorfer Strasse 1a, 1060 Vienna, Austria

**Keywords:** Clusters of orthologous genes (COG/KOG), Gene ontology (GO), KEGG pathway, Fungi, Functional enrichment analysis

## Abstract

**Background:**

The functional annotation of fungal genomes is critical for understanding their biological processes and ecological roles. While existing tools support functional enrichment analysis from publicly available annotations of well-established model organisms, few are tailored to the specific needs of the fungal research community. Furthermore, many tools struggle with processing functional annotations of novel species, for which no publicly available functional annotations are yet available.

**Results:**

FunFEA is an R package designed for functional enrichment analysis of fungal genomes. It supports COG/KOG (Clusters of Orthologous Genes), GO (Gene Ontology), and KEGG (Kyoto Encyclopedia of Genes and Genomes) annotations, and generates background frequency models from publicly available annotations for overrepresentation analysis, within a set of experimentally defined genes or proteins. Additionally, FunFEA can process eggNOG-mapper annotations, thus enabling functional enrichment analysis of novel genomes. The package offers a suite of tools for generation of background frequency models, functional enrichment analysis, as well as visualization of enriched functional categories. On release, the package includes precomputed models for 65 commonly used fungal strains in academic research and strains listed on the WHO fungal priority pathogens list.

**Conclusions:**

FunFEA fills a critical need for a specialized tool in fungal genomics, providing valuable insights into fungal biology. Additionally, its ability to process eggNOG-mapper annotations makes it an essential resource for researchers, helping to drive further exploration of fungal functional diversity and pathways and derive biological insights from novel genomes.

## Background

Functional enrichment analysis plays a pivotal role in the downstream interpretation of transcriptomics and proteomics data, enabling the identification of biological processes, molecular functions, and cellular components linked to differentially expressed genes or proteins. Several well-established annotation frameworks facilitate this process, including the eukaryotic-centric euKaryotic Orthologous Groups (KOG) [1], Gene Ontology (GO) [2], and Kyoto Encyclopedia of Genes and Genomes (KEGG) [3]. KOG offers orthology-based protein classifications, GO organizes gene products within a structured hierarchical framework, and KEGG assigns genes to known metabolic and signaling pathways, thereby enabling pathway-based analyses.

Although a variety of tools have been developed for functional enrichment analysis, they are predominantly tailored to canonical model organisms, limiting their applicability to fungal species and other non-traditional systems. This limitation is especially pressing in light of the growing recognition of fungal pathogens as a significant threat to global health. In response to this concern, the World Health Organization published its first fungal priority pathogens list in 2022 (https://www.who.int/publications/i/item/9789240060241), highlighting the urgent need for improved bioinformatic resources to advance the study of fungal biology and pathogenic mechanisms. Popular platforms such as g:Profiler [4], DAVID [5], ClusterProfiler [6], WebGestalt [7], Panther [8] and EnrichR [9] offer robust capabilities for gene set enrichment analysis; however, they are limited by the scope of their available models, typically focusing only on the most extensively studied strains of a given fungal species. Furthermore, newly sequenced or lesser-known organisms lack well-curated, comprehensive annotation databases, complicating functional enrichment analysis. This limitation restricts the ability to analyze non-reference strains or species that may harbor significant functional divergence but fall outside the coverage of these databases. To address some of these gaps, specialized fungal resources such as FungiDB [10] and FungiFun3 [11] have been developed, providing targeted support for fungal species, with FungiDB offering extensive comparative genomics tools and FungiFun3 enabling enrichment analysis based on pre-defined sets of fungal annotations. Nevertheless, both platforms are restricted to a fixed list of supported organisms and are limited in accommodating user-defined annotations from novel or uncharacterized genomes. Similarly, Flame [12] supports functional and literature-based enrichment across multiple gene sets but is reliant on external resources optimized for well-annotated model organisms, thus offering limited flexibility for custom annotations. Moreover, the aforementioned tools are limited in supporting enrichment analysis based on KOG classifications, further limiting their utility for studies relying on ortholog-based annotation frameworks commonly used in fungal genomics.

To bridge this gap, we developed FunFEA, a specialized R package for functional enrichment analyses, particularly (though not exclusively) tailored to fungal species. Designed for seamless integration into bioinformatics workflows, FunFEA can be used downstream of differential expression tools such as DESeq2 [13] or edgeR [14], enabling enrichment analysis of gene lists based on KOG, GO, and KEGG terms. It supports enrichment model generation from both public annotations, such as those available via the Joint Genome Institute (JGI) MycoCosm portal (https://mycocosm.jgi.doe.gov/mycocosm/home), and custom annotations of novel proteomes produced by eggNOG-mapper [7]. This flexibility allows researchers to perform enrichment analyses even in the absence of pre-curated reference annotations, making FunFEA especially valuable for newly sequenced, non-model, or poorly characterized organisms. By providing a unified framework for multiple annotation types and supporting ortholog-based classification, FunFEA overcomes the limitations of existing tools and broadens the scope of functional genomics analysis across both traditional and non-traditional model systems.

## Implementation

FunFEA (version 1.2.0) is an R package with minimal prerequisites and is publicly available at https://github.com/julien-charest/funfea. It requires ggplot2 [15], tidyr [16], and dplyr [17] and igraph [18] packages, and can be quickly installed by the user. A pre-built version of the package is available, featuring pre-generated enrichment models for 65 commonly studied fungal species, including those listed on the WHO fungal priority pathogens list (Table 1), based on publicly available annotations from the JGI, as well as the source code, is available for download.

**Table 1:** Available FunFEA models

Species	Strain	Source	References
*Acremonium alcalophilum*	JCM 7366	https://mycocosm.jgi.doe.gov/Acral2	[19]
*Acremonium chrysogenum*	ATCC 11550	https://mycocosm.jgi.doe.gov/Acrchr1	[20]
*Acremonium strictum*	DS1bioAY4a	https://genome.jgi.doe.gov/Acrst1	[19]
*Aspergillus fumigatus*	Af293	https://mycocosm.jgi.doe.gov/Aspfu1	[21]
*Aspergillus nidulans*	FGSC A4	https://mycocosm.jgi.doe.gov/Aspnid1	[22, 23]
*Aspergillus niger*	ATCC 1015	https://mycocosm.jgi.doe.gov/Aspni7	[24]
*Aureobasidium pullulans*	EXF-150	https://mycocosm.jgi.doe.gov/Aurpu_var_pul1	[25]
*Candida albicans*	SC5314	https://mycocosm.jgi.doe.gov/Canalb1	[26–28]
*Candida parapsilosis*	CDC317	https://mycocosm.jgi.doe.gov/Canpa1	[29]
*Candida tropicalis*	MYA3404	https://mycocosm.jgi.doe.gov/Cantro1	[30]
*Coccidioides immitis*	RS	https://mycocosm.jgi.doe.gov/Cocim1	[31]
*Coccidioides posadasii*	Silveira	https://mycocosm.jgi.doe.gov/Cocpos1	[32]
*Cryptococcus gattii*	WM276	https://mycocosm.jgi.doe.gov/CgaWM276_1	[33]
*Curvularia lunata*	m118	https://mycocosm.jgi.doe.gov/Coclu2	[34, 35]
*Fusarium acuminatum*	F829	https://mycocosm.jgi.doe.gov/Fusacu1	[36, 37]
*Fusarium avenaceum*	NRRL 54939	https://mycocosm.jgi.doe.gov/Fusave1	[36–38]
*Fusarium bataticola*	FSSC 23	https://mycocosm.jgi.doe.gov/FusspF23_1	N/A
*Fusarium camptoceras*	NRRL 13381	https://mycocosm.jgi.doe.gov/Fusca1	[36, 39–41]
*Fusarium commune*	MPI-SDFR-AT-0072	https://mycocosm.jgi.doe.gov/Fusco1	[42]
*Fusarium concolor*	NRRL 13459	https://mycocosm.jgi.doe.gov/Fuscon1	[36, 43]
*Fusarium cucurbiticola*	NRRL 22153	https://mycocosm.jgi.doe.gov/Fuscuc1	[36, 44, 45]
*Fusarium culmorum*	UK99	https://mycocosm.jgi.doe.gov/Fuscu1	[46]
*Fusarium dimerum*	NRRL 20691	https://mycocosm.jgi.doe.gov/Fusdim1	[36, 45, 47]
*Fusarium equiseti*	NRRL 66338	https://mycocosm.jgi.doe.gov/Fusequ1	[36, 40]
*Fusarium foetens*	NRRL 38302	https://mycocosm.jgi.doe.gov/Fusfoe1	[36, 48]
*Fusarium fujikuroi*	IMI 58289	https://mycocosm.jgi.doe.gov/Fusfu1	[49]
*Fusarium graminearum*	PH-1	https://mycocosm.jgi.doe.gov/Fusgr2_1	N/A
*Fusarium lateritium*	NRRL 13622	https://mycocosm.jgi.doe.gov/Fuslat1	[36, 45, 50, 51]
*Fusarium mangiferae*	MRC7560	https://mycocosm.jgi.doe.gov/Fusma1	[52]
*Fusarium mori*	NRRL 22230	https://mycocosm.jgi.doe.gov/Fusmo1	[53]
*Fusarium nisikadoi*	NRRL 25179	https://mycocosm.jgi.doe.gov/Fusnis1	[36, 50]
*Fusarium nygamai*	NRRL 66327	https://mycocosm.jgi.doe.gov/Fusnyg1	[36, 54]
*Fusarium oxysporum*	Fo47	https://mycocosm.jgi.doe.gov/FusoxFo47_2	[55]
*Fusarium poae*	NRRL 26941	https://mycocosm.jgi.doe.gov/Fuspoa1	[36, 45, 56]
*Fusarium proliferatum*	NRRL 62905	https://mycocosm.jgi.doe.gov/Fuspr1	[52]
*Fusarium pseudograminearum*	NRRL 62612	https://mycocosm.jgi.doe.gov/Fuspse1	[36, 57]
*Fusarium solani*	FSSC 5	https://mycocosm.jgi.doe.gov/Fusso1	[42]
*Fusarium sporotirchoides*	7–200	https://mycocosm.jgi.doe.gov/Fusaspor1	[58–65]
*Fusarium subglutinans*	NRRL 66333	https://mycocosm.jgi.doe.gov/Fussub1	[36, 66]
*Fusarium sulawesiensis*	NRRL 66472	https://mycocosm.jgi.doe.gov/Fussul1	[36, 41]
*Fusarium tenuicristatum*	NRRL 22470	https://mycocosm.jgi.doe.gov/Neobo1	[67]
*Fusarium tricinctum*	MPI-SDFR-AT-0068	https://mycocosm.jgi.doe.gov/Fustr1	[42]
*Fusarium vanettenii*	77–13-4	https://mycocosm.jgi.doe.gov/Necha3	[53]
*Fusarium venenatum*	MPI-CAGE-CH-0201	https://mycocosm.jgi.doe.gov/Fusven1	[42]
*Fusarium ventricosum*	NRRL 25729	https://mycocosm.jgi.doe.gov/Fusavent1	[36, 45]
*Fusarium verticilloides*	7600	https://mycocosm.jgi.doe.gov/Fusve2	[68, 69]
*Histoplasma capsulatum*	NAm1	https://mycocosm.jgi.doe.gov/Hisca1	[31]
*Lomentospora prolificans*	JHH-5317	https://mycocosm.jgi.doe.gov/Lompr1	[70]
*Madurella mycetomatis*	mm55	https://mycocosm.jgi.doe.gov/Madmy1	[71]
*Neurospora crassa*	OR74A	https://mycocosm.jgi.doe.gov/Neucr2	[72]
*Paracoccidioides brasiliensis*	Pb18	https://mycocosm.jgi.doe.gov/Parbra1	[73]
*Paracoccidioides lutzii*	Pb01	https://mycocosm.jgi.doe.gov/Parlu1	[73]
*Pichia kudriavzevii*	CBS573	https://mycocosm.jgi.doe.gov/Pickud1	[74]
*Pichia pastoris*	GS115	https://mycocosm.jgi.doe.gov/Picpa1	[75]
*Pneumocystis jirovecii*	RU7	https://mycocosm.jgi.doe.gov/Pnejir1	[76]
*Saccharomyces cerevisiae*	S288C	https://mycocosm.jgi.doe.gov/Sacce1	[77]
*Scedosporium apiospermum*	IHEM 14462	https://mycocosm.jgi.doe.gov/Sceap1	[78]
*Schizosaccharomyces pombe*	972 h-	https://mycocosm.jgi.doe.gov/Schpo1	[79, 80]
*Penicillium chrysogenum*	4,088,766	https://mycocosm.jgi.doe.gov/Pench1	[81]
*Talaromyces marneffei*	ATCC 18224	https://mycocosm.jgi.doe.gov/Talma1_2	[82]
*Trichoderma asperellum*	CBS 433.97	https://mycocosm.jgi.doe.gov/Trias1	[83]
*Trichoderma atroviride*	IMI 206040	https://mycocosm.jgi.doe.gov/Triat2	[84]
*Trichoderma reesei*	QM6a	https://mycocosm.jgi.doe.gov/Trire2	[85]
*Trichoderma reesei*	RUT C-30	https://mycocosm.jgi.doe.gov/TrireRUTC30_1	[86]
*Trichoderma virens*	Gv29-8	https://mycocosm.jgi.doe.gov/TriviGv29_8_2	[84]

## Results and discussion

### FunFEA functions

FunFEA provides several functions to build KOG, GO, and KEGG pathway models from publicly available annotations. These models are generated by grouping protein IDs based on their assigned functional categories, as defined by the annotations. This process structures the data into enrichment-ready models, allowing for efficient statistical analysis of functional categories in user-provided datasets. For KOG, the model consists of a single dataframe representing the functional categories, including the number of proteins per category and the list of proteins assigned to each category. For GO, the model is split into three dataframes representing the “biological process”, “molecular function”, and “cellular component” categories. Protein IDs are propagated up the GO hierarchy to ensure comprehensive functional representation using a graph-based tree structure [18], allowing higher-level terms to inherit annotations from their more specific descendants. For KEGG pathways, multiple models are generated by aggregating protein IDs based on pathway type, pathway class, pathway name, or enzyme definition. KEGG pathway models can also be generated using Enzyme Commission (EC) annotation if an annotation is unavailable in the KEGG database. In cases where functional annotations for a genome are not publicly available, FunFEA can generate functional enrichment models using the direct output from eggNOG-mapper, which provides functional predictions for unannotated genomes based on sequence homology from amino acid sequences.

FunFEA provides functions to calculate functional enrichment using these models and a list of proteins (e.g., from transcriptomics or proteomics data). To ensure accurate mapping, the provided protein identifiers should match those defined in the gene models of the genome assembly used to generate the enrichment annotations. Enrichment ratio is assessed by comparing the observed frequency of proteins annotated to each functional category in the input list against the background frequency of that category in the entire annotation model. By default, enrichment is computed using a one-sided hypergeometric test, with p-values adjusted for multiple testing using the Benjamini–Hochberg procedure to control the false discovery rate (FDR), yielding adjusted p-values that highlight significantly enriched categories. Alternatively, users may choose a chi-squared test for enrichment, and FunFEA supports other p-value correction methods implemented in R’s *p.adjust* method, providing flexibility in statistical analysis. To enhance interpretability, FunFEA also provides an optional redundancy reduction step for enriched GO terms. This feature identifies groups of terms that share a common ancestor within a user-defined depth in the ontology graph and retains only the most significant representative (based on p-value) from each group, leveraging the internal igraph-based GO structure in place for annotation propagation.

Additionally, FunFEA includes visualization functions to generate publication-ready enrichment plots. The plots are created as ggplot2 objects, fully customizable using the ggplot2 grammar. Users can choose between bar plots or lollipop plots to visualize enrichment results, and a wide range of customization options are available for colours, labels, and themes, allowing for a flexible and aesthetic presentation of the data. Furthermore*,* FunFEA supports integration with the patchwork package [87], enabling users to combine multiple plots into a single figure panel for efficient and cohesive presentation.

FunFEA also includes utility functions to facilitate data handling and integration. These functions can generate a conversion table linking gene names, transcript IDs, and protein IDs from GTF/GFF gene model annotations. Finally, FunFEA provides tools to convert between gene names or transcript IDs and protein IDs based on this conversion table, ensuring compatibility with enrichment analyses and other core functions and providing a seamless experience for users.

### Functional enrichment analysis with FunFEA

To demonstrate the functionality of FunFEA, we reanalyzed data from Derntl et al. (2017) in which 995 differentially expressed genes (DEGs) were identified, comparing the *xpp1* deletion strain to the QM6a wild-type parental strain [88]. Briefly, KOG and GO models were generated from functional annotations available on the JGI website (https://mycocosm.jgi.doe.gov/Trire2), and KEGG pathway models were generated from the KEGG orthology (https://rest.kegg.jp/link/ko/tre) using FunFEA core functions. First, the KOG annotation was loaded into FunFEA using the *load_kog_annotation()* function, prior to model generation using *create_kog_model()*. Enrichments were calculated with *kog_enrichment()*, using the previously generated model and a vector of protein IDs corresponding to the 995 DEGs. Similarly, GO and KEGG pathways enrichment analysis were performed using *load_go_annotation()/load_kegg_annotation()*, *create_go_model()/create_kegg_model()*, and *go_enrichment()/kegg_enrichment()* functions. Our reanalysis recapitulates the findings of Derntl et al. (2017), while extending the analysis to include functional annotations for both GO and KEGG pathways. In addition, the precision of enrichment estimates was improved by accounting for proteins mapped to multiple functional categories when calculating the number of proteins in aggregated category and the total number of annotated proteins. Of the 995 DEGs, 657 were successfully mapped to KOG terms, 604 to GO terms, and 255 to KEGG pathways.

**Fig. 1:**
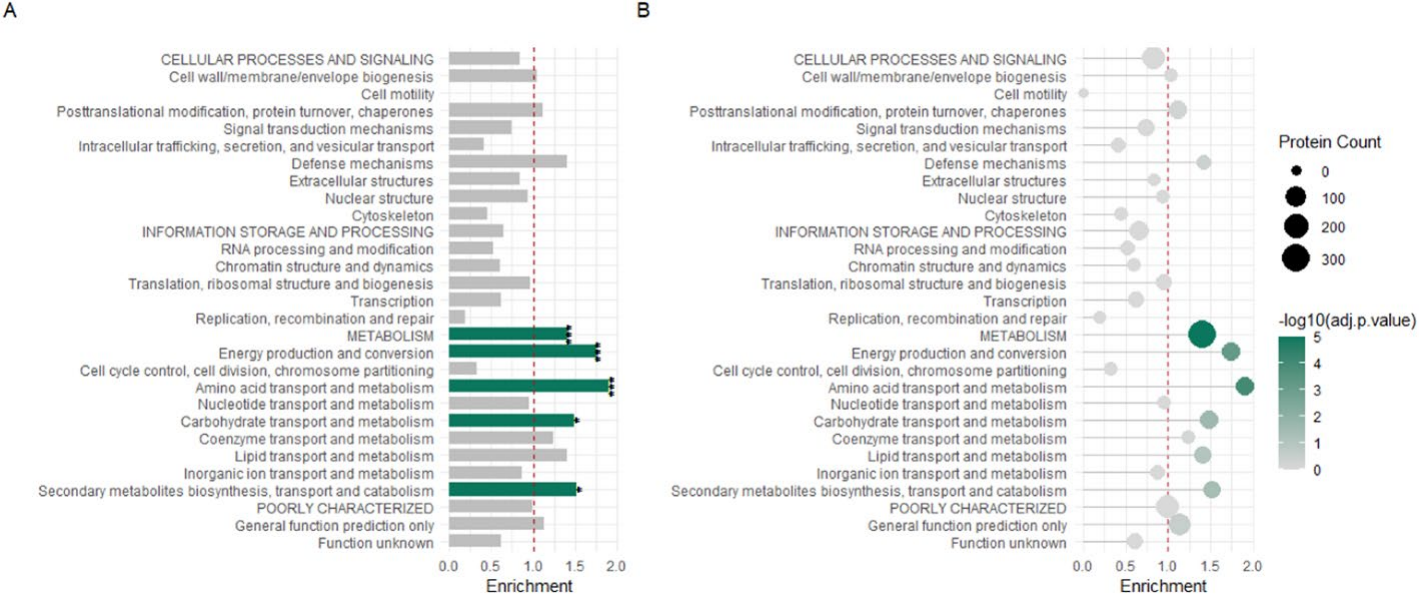
KOG enrichment analysis using FunFEA. DEGs from Derntl et al. (2017) [88] were assigned to a KOG (capitalized letters) and KOG class. Enrichment analysis compares the observed frequency of proteins in each functional category to the expected distribution using a hypergeometric test. P-values are adjusted for multiple comparisons with the Benjamini-Hochberg (BH) procedure. A) Bar plot representation of the KOG enrichment. Green bars represent KOG and KOG classes significantly enriched in the dataset. * represents a p-value smaller than 0.05. ** represents a p-value smaller than 0.01. *** represents a p-value smaller than 0.001. B) Lollipop plot representation the KOG enrichment

**Fig. 2:**
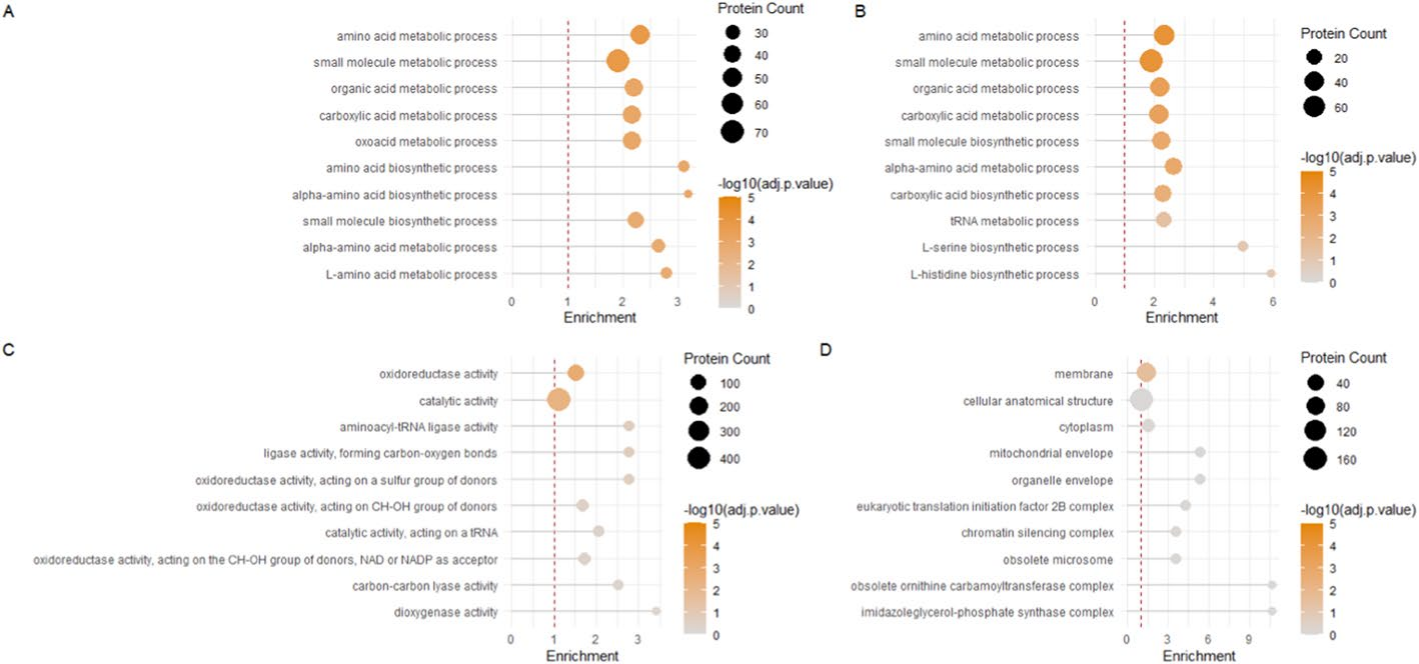
GO enrichment analysis using FunFEA. DEGs from Derntl et al. (2017) [88] were assigned to a GO term and protein IDs are propagated up the GO hierarchy to ensure comprehensive functional representation. Enrichment analysis compares the observed frequency of proteins in each functional category to the expected distribution using a hypergeometric test. P-values are adjusted for multiple comparisons with the Benjamini-Hochberg (BH) procedure. A) Lollipop plot representation of the GO term enrichment for the biological process category. B) Lollipop plot representation of the GO term enrichment for the biological process category after redundancy reduction based on graph ancestry. C) Lollipop plot representation of the GO term enrichment for the molecular function category. D) Lollipop plot representation of the GO term enrichment for the cellular component category

**Fig. 3:**
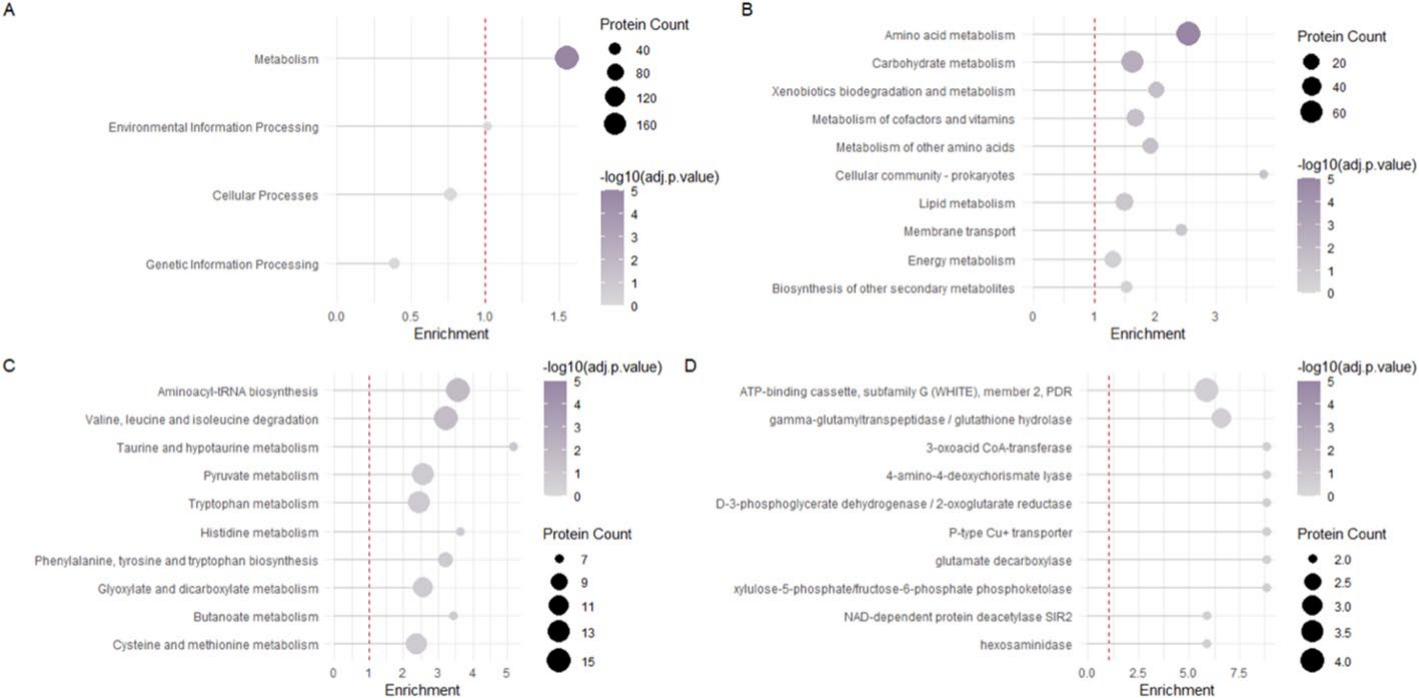
KEGG pathway enrichment analysis using FunFEA. DEGs from Derntl et al. (2017) [88] were assigned to a KEGG orthology. Enrichment analysis compares the observed frequency of proteins in each functional category to the expected distribution using a hypergeometric test. P-values are adjusted for multiple comparisons with the Benjamini-Hochberg (BH) procedure. A) Lollipop plot representation of the KEGG pathway type enrichment. B) Lollipop plot representation of the KEGG pathway class enrichment. C) Lollipop plot representation of the KEGG pathway name enrichment. D) Lollipop plot representation of the KEGG enzyme definition enrichment

We present several enrichment plots to illustrate the results of functional enrichment analysis using FunFEA, generated using the *generate_kog_plot()*, *generate_go_plot()* and *generate_kegg_plot()* functions. For KOG enrichment (Fig. 1), we identify overrepresented categories associated with energy production and conversion, amino acid transport and metabolism, carbohydrate transport and metabolism, as well as secondary metabolite biosynthesis, transport and catabolism, highlighting the major metabolic functions impacted in the dataset. GO enrichment (Fig. 2) reveals a significant overrepresentation of processes involved notably in amino acids and organic acid metabolic biological processes, as well oxidoreductase activity molecular function. No significant enrichment has been observed in cellular components. KEGG pathway enrichment (Fig. 3) highlights metabolic pathways, including amino acid metabolism and carbohydrate metabolism.

To demonstrate FunFEA’s compatibility with annotations generated by eggNOG-mapper, the complete proteome of *Trichoderma reesei* (QM6a) was retrieved from the JGI MycoCosm portal (https://mycocosm.jgi.doe.gov/Trire2) and functionally annotated using eggNOG-mapper (v2.1.12) with the eggNOG database (v5), employing DIAMOND for sequence alignment and restricting the taxonomic scope to fungi, with a e-value threshold of 0,001. Briefly, the eggNOG-mapper annotation was loaded into FunFEA using the *load_eggnog_annotation()* function, and KOG, GO and KEGG models were generated using the *create_kog_model_eggnog()*, *create_go_model_eggnog()* and *create_kegg_model_eggnog()* functions, respectively. Data from Derntl et al. (2017) was reanalyzed using these eggNOG-derived models and, of the 995 DEGs, 785 were successfully mapped to KOG terms, 341 to GO terms, and 264 to KEGG pathways. Enrichments and figures were generated as previously described for publicly available annotations.

**Fig. 4:**
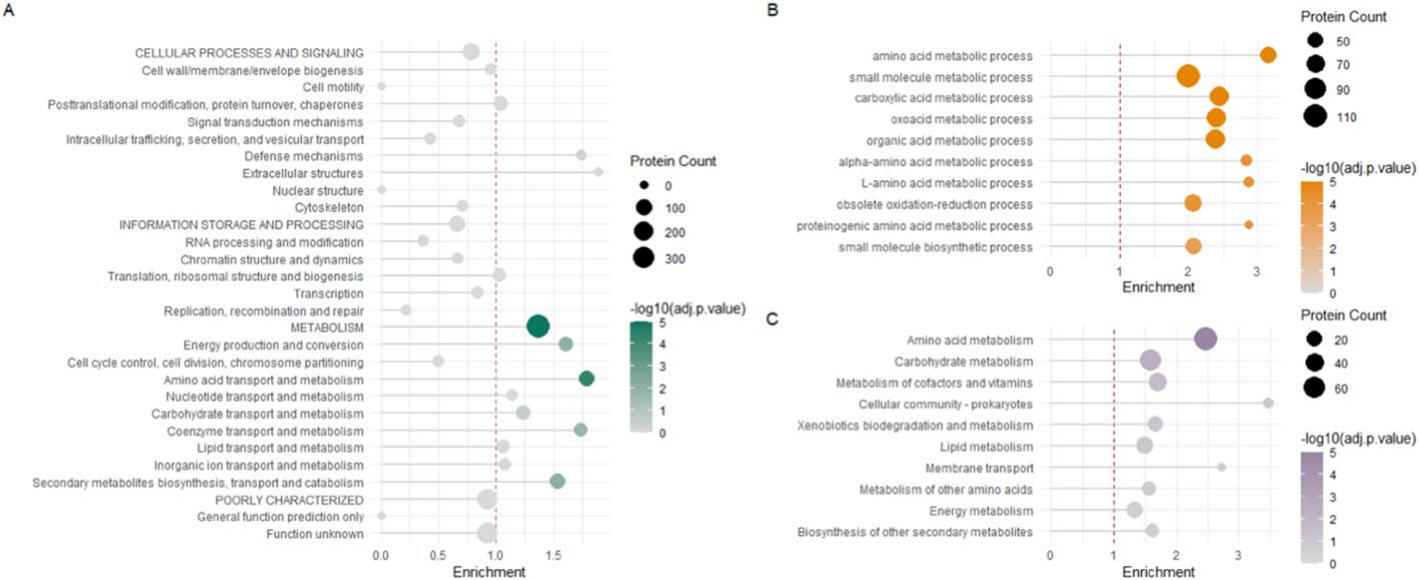
KOG, GO and KEGG pathway enrichment analysis using FunFEA’s eggNOG-derived models. DEGs from Derntl et al. (2017) [88] were assigned to a KOG, GO and KEGG orthology based on functional prediction by eggNOG-mapper. Enrichment analysis compares the observed frequency of proteins in each functional category to the expected distribution using a hypergeometric test. P-values are adjusted for multiple comparisons with the Benjamini-Hochberg (BH) procedure. A) Lollipop plot representation of the KOG enrichment. B) Lollipop plot representation of the GO term enrichment for the biological process category. C) Lollipop plot representation of the KEGG pathway class enrichment

We present enrichment plots to illustrate the results of functional enrichment analysis using FunFEA models derived from eggNOG-mapper annotations. For KOG enrichment (Fig. 4A), we identify overrepresented categories associated with energy production and conversion, amino acid transport and metabolism, coenzyme transport and metabolism, as well as secondary metabolite biosynthesis, transport and catabolism, recapitulating the results obtained from curated JGI annotations, with the main difference being carbohydrate transport and metabolism falling under the significance threshold after p-value correction. Additionally, GO enrichment (Fig. 4B) and KEGG pathway enrichment (Fig. 4C) recapitulate results obtained from curated annotations.

To facilitate reproducibility and provide users with a practical example, we have included an example folder in the GitHub repository containing all necessary input files to test the package and reproduce the analyses and figures presented. This includes the list of protein IDs, gene models, KOG, GO, and KEGG annotations obtained from JGI, KEGG pathway mappings retrieved from the KEGG database, the corresponding proteome sequences, and functional annotations derived from eggNOG-mapper. Complete workflows are also provided within the example folder, guiding users through each step of the analysis using these data.

### Limitations

FunFEA does not currently support internal conversion between identifiers from different databases or annotation systems (e.g., Ensembl, RefSeq, UniProt, InterPro). Protein identifiers provided by the user must exactly match those used in the functional annotation model, requiring external preprocessing when working with datasets based on alternative nomenclatures. Additionally, due to variability in FASTA header formats across different proteome files, users may need to adjust the query identifiers in the eggNOG-mapper output to match the protein IDs, transcript IDs or gene names retrieved from upstream analysis. This can, however, be performed seamlessly within FunFEA and we provide an example in the related example workflow.

## Conclusion

FunFEA provides a dedicated framework for functional enrichment analysis tailored to fungal and non-model organisms, enabling researchers to uncover the biological relevance of transcriptomic and proteomic datasets. By bridging the gap between genome-wide functional annotation and enrichment workflows, FunFEA broadens the accessibility of these approaches beyond classical model systems, facilitating meaningful interpretation even for newly sequenced or sparsely annotated genomes. The package currently includes 65 precomputed models for KOG, GO, and KEGG enrichment, selected to reflect species of interest to the fungal research community and the World Health Organization (WHO), with plans to expand this collection over time. Additionally, FunFEA offers flexible tools to generate enrichment models from publicly available annotations on the JGI MycoCosm portal or from eggNOG-mapper outputs, supporting custom analyses on novel or poorly characterized genomes.

### Availability and requirement

Project name: FunFEA. Project home page: https://github.com/julien-charest/funfea. Operating system: Platform independent. Programming language: R. Other requirements: R > = 2.10. Dependencies: ggplot2, tidyr, dplyr, igraph. License: GPL-2. Any restriction to use by non-academics: none.

## Data Availability

The source code, as well as a compiled version of FunFEA (version 1.2.0) is available at https://github.com/julien-charest/funfea. Publicly available functional annotations used to build the models (Table 1) were obtained from the Joint Genome Institute (JGI). Differentially expressed genes identified in Derntl et al. [88] were used in this article to demonstrate the functionality of FunFEA.

## Abbreviations

COG: Clusters of orthologous genes
DEG: Differentially expressed gene
GO: Gene ontology
KEGG: Kyoto encyclopedia of genes and genomes
KOG: EuKaryotic orthologous groups
